# Warburg-associated acidification represses lactic fermentation independently of lactate, contribution from real-time NMR on cell-free systems

**DOI:** 10.1038/s41598-023-44783-3

**Published:** 2023-10-18

**Authors:** Zoé Daverio, Maxime Kolkman, Johan Perrier, Lexane Brunet, Nadia Bendridi, Corinne Sanglar, Marie-Agnès Berger, Baptiste Panthu, Gilles J. P. Rautureau

**Affiliations:** 1 Laboratoire CarMeN, UMR INSERM U1060/INRAE U1397, University of Lyon, Université Claude Bernard Lyon 1, 69310 Pierre-Bénite, France; 2 Master de Biologie, École Normale Supérieure de Lyon, University of Lyon, Université Claude Bernard Lyon 1, 69342 Lyon Cedex 07, France; 3 Institut de Chimie et Biochimie Moléculaires et Supramoléculaires, ICBMS UMR 5246, University of Lyon, Université Claude Bernard Lyon 1, 69622 Lyon, France; 4 Institut des Sciences Analytiques, UMR5280 CNRS, University of Lyon, Université Claude Bernard Lyon 1, 5 rue de la Doua, 69100 Villeurbanne, France

**Keywords:** Cancer metabolism, Cancer microenvironment

## Abstract

Lactate accumulation and acidification in tumours are a cancer hallmark associated with the Warburg effect. Lactic acidosis correlates with cancer malignancy, and the benefit it offers to tumours has been the subject of numerous hypotheses. Strikingly, lactic acidosis enhances cancer cell survival to environmental glucose depletion by repressing high-rate glycolysis and lactic fermentation, and promoting an oxidative metabolism involving reactivated respiration. We used real-time NMR to evaluate how cytosolic lactate accumulation up to 40 mM and acidification up to pH 6.5 individually impact glucose consumption, lactate production and pyruvate evolution in isolated cytosols. We used a reductive cell-free system (CFS) to specifically study cytosolic metabolism independently of other Warburg-regulatory mechanisms found in the cell. We assessed the impact of lactate and acidification on the Warburg metabolism of cancer cytosols, and whether this effect extended to different cytosolic phenotypes of lactic fermentation and cancer. We observed that moderate acidification, independently of lactate concentration, drastically reduces the glucose consumption rate and halts lactate production in different lactic fermentation phenotypes. In parallel, for Warburg-type CFS lactate supplementation induces pyruvate accumulation at control pH, and can maintain a higher cytosolic pyruvate pool at low pH. Altogether, we demonstrate that intracellular acidification accounts for the direct repression of lactic fermentation by the Warburg-associated lactic acidosis.

## Introduction

The metabolic signature of cancer has long been associated with the Warburg effect, defined by the hyperactivity in aerobic conditions of lactic fermentation, i.e. the conversion of glucose into lactate^1^. Although initially described in cancer cells, Warburg-like metabolism has also been observed in proliferative cells with high energetic needs^2^. Because of its low ATP yield, this signature stands as a paradox^2–5^. Its widespread association to cancer and malignancy has motivated several hypotheses to explain the metabolic benefits associated with the Warburg effect. For example, at the scale of individual cells, lactic fermentation could provide anabolic intermediates at a high rate and with fewer regulatory constraints than mitochondrial metabolism, and maintain cellular redox balance when mitochondrial NADH shuttles are outpaced^6^. At the whole-tumour scale, the Warburg effect is associated with lactic acidosis, which consists in high lactate concentrations and low pH in the tumour microenvironment, as a result of lactate and proton secretion^7^. Lactic acidosis has been shown to benefit tumours in numerous ways, for example: by suppressing immune cell activity^8^, by promoting aggressive phenotypes^9^, and by rewiring cancer cell metabolism to help their survival under nutritional stress^7,10^. In particular, lactic acidosis enables the resistance to glucose depletion^7^, a condition resulting from glucose overconsumption and poor vascular replenishment in tumours^11^. Strikingly, Wu et al.^12^ evidenced that lactic acidosis in a glucose-deprived culture medium increased from 1 to 10 days the survival time of 90% cells. They later showed that lactic acidosis downregulates lactic fermentation and promotes a more economical use of glucose: extracellular lactic acidosis resulted in cytosolic acidification and lactate accumulation, slowed glucose consumption down to one third of its initial rate, reversed lactate production in a consumption, and reactivated mitochondrial ATP production^13,14^. The same authors suggested that intracellular acidification contributed to the rewiring of glucose catabolism by inhibiting lactic fermentation^13^.

We and others have demonstrated that cell-free systems (CFS) are well suited to study metabolic processes that are specific to a single cell compartment, such as lactic fermentation in the cytosol. CFS consist of isolated cellular compartments complemented with appropriate reactants, metabolic substrates and ATP sources that allow the reactivation of metabolic activities^15^. On the one hand, CFS present the complexity of the biological systems they originate from, with the conserved enzyme content and functionality that are necessary to the study of metabolic pathways such as glucose catabolism^16,17^. On the other hand, they enable the direct alteration of compartment parameters such as lactate concentration and pH, as they abrogate compartmentation effects such as nutrient uptake, interorganelle metabolite fluxes and pH regulations^15,18^. Real-Time Nuclear Magnetic Resonance (RT-NMR) enables the monitoring of multiple compound evolutions over various timescales in a complex system. Applied to CFS, RT-NMR gives precise insights over numerous enzymatic reactions occurring simultaneously in an isolated and active cellular compartment^15,19–21^.

In this study we used RT-NMR to monitor the metabolism of cytosolic CFS and assess how acidification and lactate accumulation impact glucose catabolism in the context of cancer. The input pH was adjusted from 7.6 to 6.5 to span over the widest range encountered in cancer^22^ and the input lactate concentration ranged from its intrinsic value in the CFS of ~ 3–40 mM, beyond the physiological maximum of ~ 23 mM^23^. The readouts of cytosolic glucose catabolism were the evolutions of glucose, lactate, and pyruvate. We found that acidification strongly represses lactic fermentation whereas lactate supplementation alters pyruvate homeostasis in CFS originating from the proliferative and Warburg-type Human Embryonic Kidney (HEK) cell line. To determine whether these effects were specific to this proliferative Warburg phenotype, experiments were replicated with CFS prepared from cell lines showing different phenotypes of cancer and lactic fermentation: the HeLa cell line, which present a Warburg-type cancer phenotype, the INS-1E insulinoma cell line with no Warburg effect^24^, and rabbit reticulocytes that are non-cancer cells without mitochondria^25^ relying on lactic fermentation for ATP production^26–28^. The effects of acidification found in the HEK model were confirmed in HeLa and reticulocytes, suggesting that acidification impacts different phenotypes of lactic fermentation in a similar fashion. In contrast, the effects of high lactate concentration highly depended on the CFS model.

## Material and methods

### Commercial cytosols, cell lines and culture

HeLa cytosols (88882) and Rabbit Reticulocyte lysates (L4540) were respectively purchased from Thermofisher and Promega. Human Embryonic Kidney (HEK) and rat insulinoma INS-1E cell lines were respectively obtained from ATCC and MTA, Professor Maescler’s laboratory (RRID:CVCL_0351). Both cell lines were routinely maintained in our laboratory. HEK cells were maintained in DMEM (Gibco) media with 25 mM glucose, 1 mM pyruvate, 2 mM Glutamax (Gibco), 10% SVF, and 1% penicillin/streptomycin (Dutscher). INS-1E cells were maintained in RPMI 1640 (Gibco) with 1 mM pyruvate, 2 mM Glutamax, 10 mM HEPES buffer, 50 µM β-mercaptoethanol, 5% SVF, and 1% penicillin/streptomycin. % refer to volume proportions.

### Cell lysis and subcellular fractionation

Cytosolic fractions were prepared as previously reported^29^. Cells were scraped, centrifuged at 300 g for 5 min, washed with PBS buffer, resuspended in an isovolume of R buffer (10 mM HEPES pH 7.4, 10 mM CH_3_CO_2_K, 1 mM (CH_3_CO_2_)_2_ Mg and 1 mM DTT) and mechanically lysed using a Dounce homogeniser (Pestle B). The lysis was checked by microscopy. The lysate was then centrifuged at 16,000 g for 5 min before the supernatant was collected as the cytosolic fraction. The pellet, that contained nuclei, mitochondria and plasma membranes, was resuspended in an isovolume of R buffer and centrifuged at 300 g for 5 min. The supernatant containing mitochondria was then rinsed 3 times with R buffer. Cytosolic and mitochondrial fractions were frozen separately and stored at − 80 °C.

### Protein quantification and Western Blot

The total protein concentration in cytosolic and mitochondrial fractions was determined by BCA assay (Bio-Rad). 20 µg of samples were denatured in Laemmli buffer at 75 °C for 10 min. They were then resolved on a 10% Sodium Dodecyl Sulfate Polyacrylamide gel (SDS-PAGE), and transferred to a Polyvinylidene fluoride (PVDF) membrane. SDS-PAGE gels were stained with Coomassie blue. Membranes were blotted using anti-VDAC-1 (sc8828, Santa Cruz Biotechnology), -GAPDH (#AC024, ABclonal), -LDH-A, -LDH-B (HPA019007, Atlas Antibodies), -phosphoLDH-A, -PFK-FB2 (#13045S, Cell Signaling), pPFK-FB2 (#13064S, Cell Signaling), -aldolase A (#3188S, Cell Signaling), PKM1/2 (#3106S, Cell Signaling), -HK-1 (#2024, Cell Signaling), -HK-2 (#A0994, Cell Signaling), -HK-4 (#3782, Cell Signaling), -α-Tubulin (#TS168, Cell Signaling). For each blot the Precision Plus Protein Dual Color Standard (Bio-Rad) was used as molecular ladder.

### Equipment and settings

Western Blot image acquisition was performed using the Bio-Rad ChemiDoc MP Imaging System, using chemiluminescence and colorimetric modes for revealing antibodies and molecular ladder, respectively. Chemiluminescent and colorimetric pictures of the same membrane were merged using the Bio-Rad Image Lab software.

### In vitro translation assay

All used cytosols were complemented with 25 μM Hemin (Fluka), 50 mg/L of creatine kinase (Roche), 50 mg/L of bovine liver tRNA (Sigma–Aldrich) and their functionality was checked by an in vitro translation test. Complemented cytosols were mixed with an isovolume of reaction mix containing 10 mM phosphocreatine (Fluka), 75 mM CH_3_CO_2_K, 0.75 mM (CH_3_CO_2_)_2_Mg, 2 mM D-glucose (Sigma-Aldrich), 20 µM amino acids (Sigma-Aldrich), and 100 ng homemade EMCV-HiBiT-GFP-coding mRNA and incubated at 30 °C for 30 min^29,30^. The corresponding protein was detected with the Nano-Glo© HiBiT Lytic Detection System (Promega).

### Sample preparation for RT-NMR

Cytosols (100 µL) were mixed with an isovolume of reaction mix, giving a final volume of 200 µL CFS containing 10 mM phosphocreatine, 75 mM KCl, 0.75 mM MgAcetate, 20 µM aminoacid mix, 5 µg creatine phosphate kinase (Roche), 5 mM imidazole, 1 µg homemade EMCV-HiBiT-GFP-coding mRNA, 10% D_2_O, various concentrations of lactate (0, 5, 10, 15, 20, 25, 40 mM) and 40 mM of HEPES or PIPES buffers adjusted at various pH. CFS were incubated for 30 min at 30 °C, supplemented with 1 mM glucose and immediately transferred to a 3 mm diameter NMR tube. NMR acquisition started between 9 and 15 min after glucose addition.

### NMR acquisition

All NMR experiments were conducted on a Bruker AVANCE III HD 600 MHz spectrometer equipped with a TXI probe, 5 mm tube, H-C/N detection and a z-gradient. The temperature was maintained at 30.0 °C and the autoshim function activated throughout all experiments. We used the Bruker pulse sequence cpmgpr1d, a one-dimensional ^1^H experiment with water suppression and a Carr-Purcell-Meiboom-Gill (CPMG) filter. This sequence was chosen to focus on the small metabolites present in CFS, concealing the background signals of large molecules such as lipids, proteins and nucleic acids. For each spectrum, 32 scans were performed, each one with an acquisition step lasting 0.9998 s and recording 16,804 points, and the relaxation delay D1 was set to 4 s. The total experiment time for one spectrum equalled 3 min and 4 s. For the RT monitoring, successive spectra were acquired from 80 min to 2 h, corresponding to 30–40 spectra. Some samples were assessed for longer periods, up to 300 spectra, thus up to 15 h. Spectra were calibrated by setting the glucose doublet at 5.23 ppm, automatically phased, baseline-corrected and integrated. These steps were achieved on TopSpin Bruker software (Bruker inc.).

### Metabolite concentration and pH monitoring

A lactate standard solution (1.0 g/L, Fisher) served as external calibration for the quantification of lactate (integration of the doublet at 1.3 ppm). All NMR spectra were exploited via the ERETIC2 utility from TopSpin to add a digitally synthesised signal to all spectra^31^, allowing the use of ChenomX NMR Suite 8.6 (*ChenomX Inc., Edmonton, Canada*) according to the manufacturer’s procedure. Eretic linewidth was adjusted to fit the alanine peak at 1.46 ppm, and unambiguous signals were selected for the quantifications of phosphocreatine (or creatine phosphate, singlet at 3.94 ppm), creatine (singlet at 3.92 ppm), glucose (doublet at 5.23 ppm), and pyruvate (singlet at 2.37 ppm, overlapping with glutamate signals which were therefore also fitted). PH was monitored after the chemical shift of the ~ 8.1 ppm imidazole pH-sensitive peak^32^.

### Statistical analysis

Statistical analyses were performed with the R software. Correlations between compound concentration and time were tested either with a Pearson's correlation test, for data that had a normal dispersion around a linear regression model, and no outliers, or a Spearman's correlation test for the data not meeting these conditions. The significance threshold was taken as *p*-value < 0.05.

## Results

### RT-NMR on CFS allows to monitor the glucose catabolism of metabolically functional cytosols in controlled conditions

**Figure 1:**
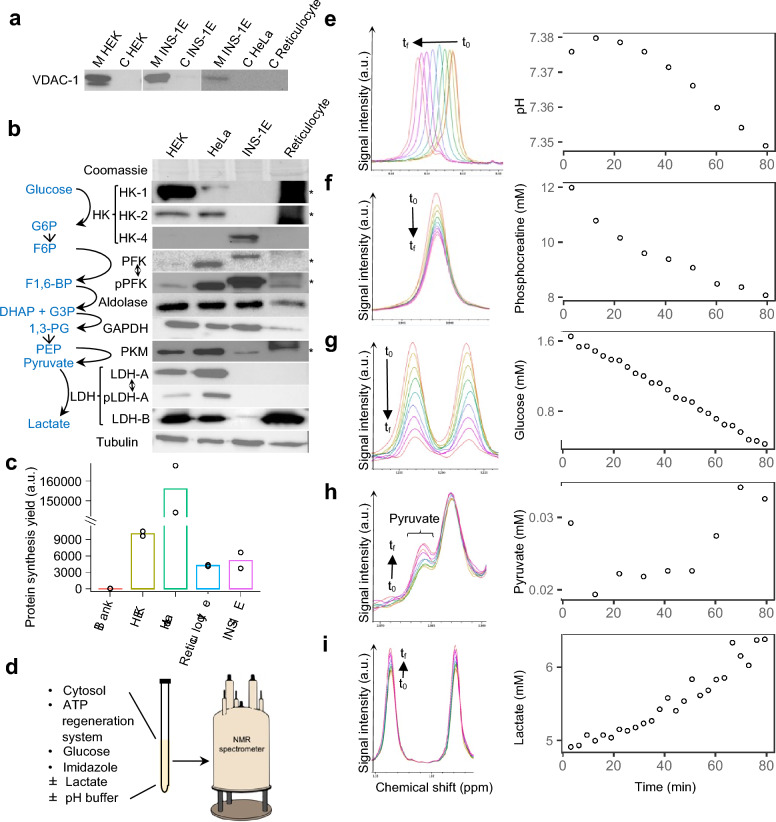
Experimental workflow of RT-NMR analysis of CFS. (**a**) Western Blot analysis of the mitochondrial voltage-dependent anion channel 1 (VDAC-1) in the cytosolic (C) and mitochondrial (M) fractions of HEK and INS-1E cells, and in commercial HeLa and reticulocyte cytosols. Original blots and gel are available in Supplementary Fig. S1. (**b**) Western Blot analysis of cytosolic fractions of HEK, INS-1E, HeLa cells and rabbit reticulocytes. The following enzymes were revealed: hexokinase isoforms (HK-1, -2 and -4), phosphofructokinase (PFK) and phosphorylated PFK (pPFK), aldolase, glyceraldehyde-3-phosphate dehydrogenase (GAPDH), pyruvate kinase M (PKM), lactate dehydrogenase isoforms (LDH-A and -B) and phosphorylated LDH-A (pLDH-A). Coomassie gel staining and tubulin served as loading controls. A parasite band (*) spanning from 75 to 150 kDa was observed in reticulocyte cytosols. Single-ended arrows indicate a conversion between metabolites (in blue). Double-ended arrows indicate enzyme post-translational modifications. Brackets regroup the different isoforms of a single enzyme. Original blots and gel are available in Supplementary Fig. S2. (**c**) In vitro translation test on HEK, HeLa, reticulocyte and INS-1E CFS. (**d**) CFS composition: cytosols were complemented with an ATP regeneration system, glucose, imidazole and adjusted at variable lactate concentrations and pH. (**e**–**i**) In RT-NMR time series over 80 min (from an initial time t_0_ to a final time t_f_), individual compounds were associated with a number of peaks. The left side of the panels correspond to overlaid NMR peaks differing only by acquisition time. The right side of the panels correspond to the pH (**e**) and the compound concentrations (**f**–**i**) in the CFS obtained from NMR time series. The chemical shift of the imidazole peak allowed to determine the pH of the CFS over time (**e**); the peak area, proportional to the concentration of the affiliated molecules, allowed to quantify phosphocreatine (**f**), glucose (**g**), pyruvate (**h**) and lactate (**i**) over time.

The quality of the four studied cytosols (HEK, HeLa, INS-1E and reticulocyte cytosols) was assessed by Western Blot analysis and translation assays. No cytosol presented the VDAC-1 protein, a mitochondrial marker, which indicated that they were devoid of mitochondria (Fig. 1a). For reticulocyte cytosols, a parasite band prevented the detection of proteins of size between 75 and 150 kDa (Fig. 1b). All cytosols contained the tested and detectable enzymes of lactic fermentation, namely hexokinase (HK), phosphofructokinase (PFK) and phosphorylated PFK (pPFK), aldolase, glyceraldehyde-3-phosphate dehydrogenase (GAPDH), pyruvate kinase M (PKM), and lactate dehydrogenase (LDH). The HK and LDH isoforms differed between cytosols. The HK-1 and -2 isoforms were abundant in HEK and HeLa cytosols, whereas the HK-4 isoform was specific to the INS-1E cytosols. HEK and HeLa cytosols presented abundant LDH-A and -B, and phosphorylated LDH-A (pLDH-A), whereas INS-1E and reticulocyte cytosols presented only LDH-B, in low and high abundance respectively. Control in vitro translation assays attested that cytosols could achieve protein synthesis (Fig. 1c), one of the most complex and ATP-consuming metabolic processes in the cytosol^29,30^. Moreover, we already described that in the presence of an ATP regeneration system, ATP and GTP endogenous levels were maintained. As the NMR sample contains 50% of cytosol, we estimate that ATP and GTP levels are 50% of the physiological ones^15^.

For the study of lactic fermentation by RT-NMR, the metabolic activity of cytosols was reactivated by the addition of an ATP regeneration system (phosphocreatine and creatine kinase) and glucose (Fig. 1d). Multiple and simultaneous metabolite evolutions were followed by RT-NMR that yielded 80-min time series. Following the pH-dependent chemical shift of the 8.1 ppm imidazole peak allowed the determination of the initial pH and the monitoring of pH fluctuations (Fig. 1e). The decrease in phosphocreatine (Fig. 1f) indicated that CFS were metabolically active. The detectable metabolites of glucose catabolism were glucose, pyruvate and lactate (Fig. 1g–i respectively). All in all, RT-NMR enabled us to monitor the pH and several reactions of glucose catabolism in metabolically functional CFS showing active translation and ATP consumption.

### Acidification slowed glycolysis down and halted lactate production in HEK CFS

**Figure 2:**
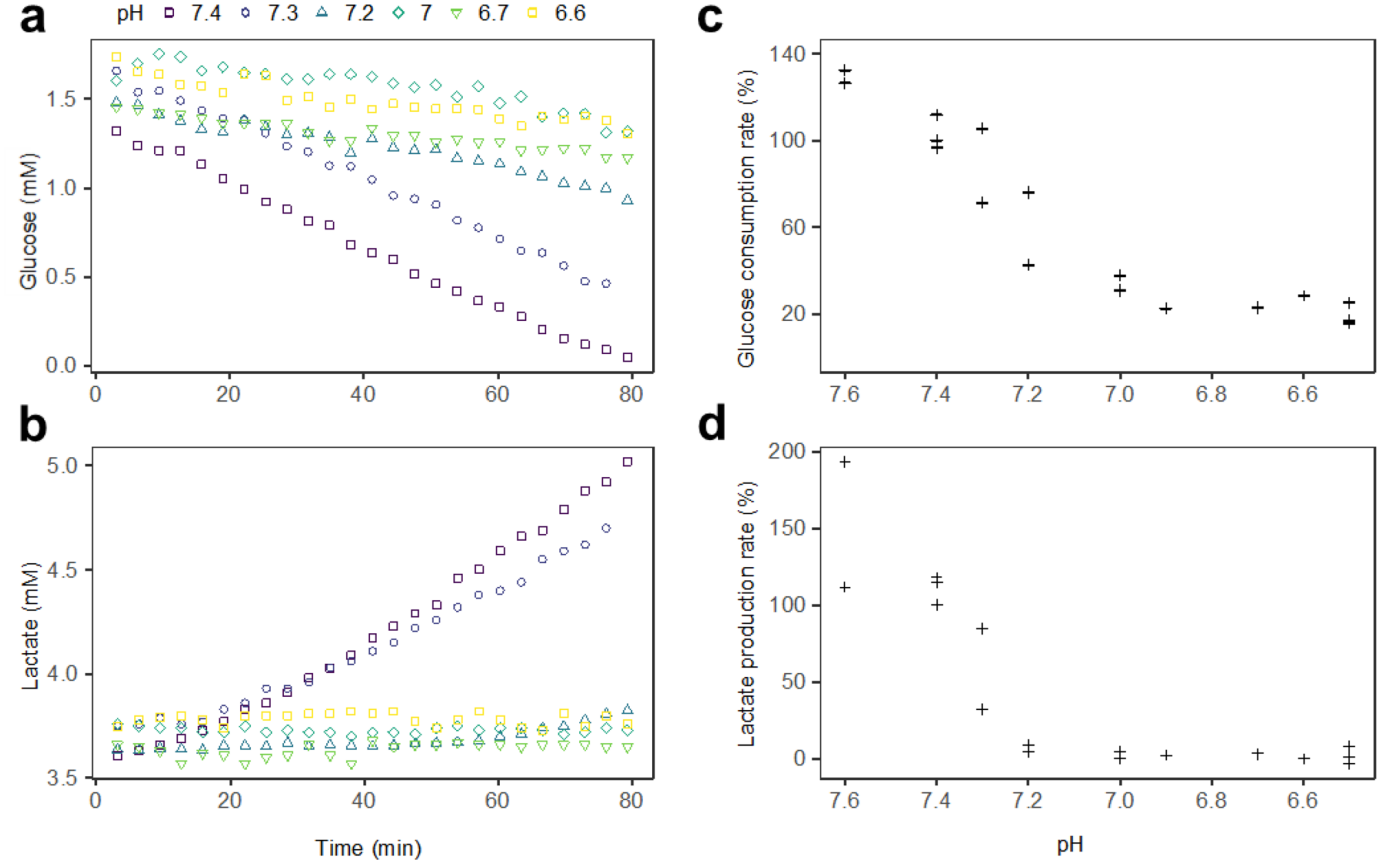
Acidification slowed glucose consumption down and inhibited lactate production in HEK CFS. (**a**, **b)** Overlaid 80-min glucose (**a**) or lactate (**b**) evolutions in a single biological replicate of HEK CFS at pH 7.4 (), 7.3 (), 7.2 (), 7 (), 6.7 () or 6.6 (). (**c**, **d**) Corresponding rates of glucose consumption (**c**) and lactate production (**d**) normalised to the control condition (pH 7.4) for HEK CFS replicates.

HEK CFS were employed to investigate how a Warburg-type glucose catabolism reacted to a progressive decrease in pH. A continuum of pH values between 7.6 and 6.5 was explored. In control conditions, the unadjusted CFS pH was between 7.4 and 7.5. The initial lactate concentration was around 3 mM in all pH conditions. The consumption rate of phosphocreatine, indicating the activity of ATP-consuming processes in the CFS, was not impacted by pH (Supplementary Fig. S3). We could thus assess how acidification specifically impacts the simultaneous evolutions of glucose (Fig. 2a) and lactate (Fig. 2b). In every run, glucose was consumed in a linear fashion until its complete depletion. Lactate either remained constant or accumulated linearly until glucose depletion, when lactate concentration reached its plateau. In the control condition (pH 7.4) glucose was consumed at a rate of 17 µM/min and lactate was produced at a rate of 19 µM/min. With acidification from pH 7.4 to 7.2, glucose consumption was progressively slowed down and lactate production became undetectable. Under pH 7.2, glucose consumption was maintained at a low rate and lactate production remained undetectable.

Figure 2c and d respectively display the rates of glucose consumption and lactate production, normalised to the control condition of pH 7.4, for several HEK CFS replicates. The glucose consumption rate decreased drastically with acidification until pH 7 where it equalled 31–38% of its value in control conditions (Fig. 2c). Below pH 7 the relative glucose consumption rate was maintained at 16–31%. At a slightly alkaline pH of 7.6 the glucose consumption rate was increased to 126–132%. The lactate production rate steeply decreased from 112 to 194% at pH 7.6 to 4–9% at pH 7.2 (Fig. 2d). Under pH 7.2, production rates were oscillated randomly around 0%, between − 3% and 8%.

Below pH 7.2 the cancellation of lactate production along with the maintenance of a low-rate glucose consumption, indicated that the glucose consumption and its specific conversion into lactate were both inhibited.

### High lactate concentration impacted neither glucose consumption nor lactate production, but promoted pyruvate accumulation in HEK CFS

HEK CFS were employed to investigate how a Warburg-type glucose catabolism reacted to a progressive lactate accumulation. The tested lactate concentration ranged between ~ 3 mM, which was the intrinsic content of CFS, to 40 mM, which exceeded the maximal value reported in cancer^23^. Lactate addition to the CFS affected neither pH, which remained at 7.4 at all lactate concentrations, nor the consumption rate of phosphocreatine (Supplementary Fig. S3).

**Figure 3:**
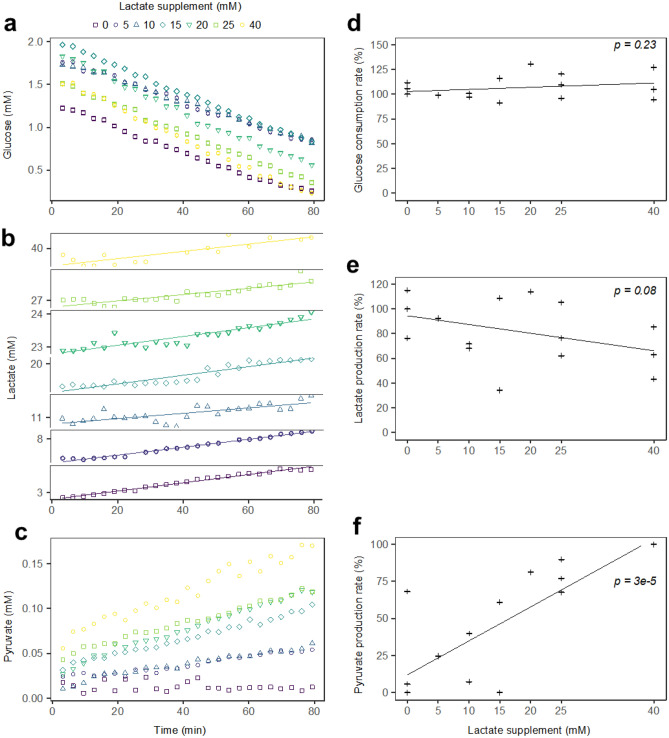
High lactate concentration impacted neither glucose consumption nor lactate production significantly, but promoted pyruvate accumulation in HEK CFS. (**a–c**) Overlaid 80-min glucose (**a**), lactate (**b**) and pyruvate (**c**) evolution for a single HEK CFS supplemented with 0 (), 5 (), 10 (), 15 (), 20 (), 25 () or 40 () mM lactate. In the (**b**) panel, all boxes have the same y-axis scale. (**d**–**f**) Rates of glucose consumption (**d**), lactate production (**e**) and pyruvate accumulation (**f**) normalised to the control condition (**d**, **e**) or to the 40 mM lactate condition (**f**). p-values are the results of Pearson’s correlation tests. Indicative trendlines are the results of linear regression models.

The evolutions of glucose, lactate and pyruvate (Fig. 3a–c respectively) were monitored over the tested range of lactate concentration (3–40 mM). Glucose and lactate evolutions did not seem affected by an increase in lactate concentration. Interestingly, pyruvate concentration remained constant around 0.015 mM in the control condition, whereas with lactate supplementation pyruvate accumulated in a well-defined, dose-dependent fashion. Pyruvate accumulated up to 0.054 mM with a 5 mM lactate supplementation and up to 0.17 mM with a 40 mM lactate supplementation.

Figure 3d and e respectively show the rates of glucose consumption and lactate production normalised to the control condition, as a function of lactate concentration, for several HEK CFS replicates. No significant correlation was found between the normalised glucose consumption rate and the lactate concentration (Fig. 3d, p-value = 0.23). With increasing lactate concentration, the lactate production rate tended to decrease from 76 to 115% without lactate supplementation to 43–85% with 40 mM lactate, but this decrease was not significant (Fig. 3e, p-value = 0.08). Figure 3f shows the rate of pyruvate accumulation, relative to its maximum with a lactate supplementation of 40 mM. A significant correlation between lactate concentration and pyruvate accumulation rate can be observed (p-value = 3e−5). Altogether, the addition of lactate neither impacted the glucose consumption nor significantly decreased the lactate production capacity of HEK CFS, but caused a dose-dependent rise in pyruvate concentration, which otherwise remained constant in the control condition.

### The effect of acidification dominates the one of lactate in different lactic fermentation phenotypes

The present section addresses how the effects of acidification and lactate accumulation interact together and whether they could be conserved across diverse phenotypes of cancer or lactic fermentation. To this end, CFS obtained from three more cell types were employed (Supplementary Table S1). The HeLa cell line, similarly to the HEK one, displays a proliferative phenotype along with a strong Warburg effect^33^; the INS-1E insulinoma cell line is cancer-type but less proliferative than HEK and HeLa cells, and does not consume glucose, thus shows no Warburg effect^24^; rabbit reticulocytes are non-cancer cells performing lactic fermentation^26–28^ (Supplementary Table S1). In control conditions the four CFS showed different initial pH: 7.4–7.5 for HEK, 6.9 for HeLa, 7.3 for reticulocyte, and 7.5 for INS-1E (Supplementary Table S1). All CFS were submitted to four conditions: control, adjustment to low pH (from 6.4 to 6.8 depending on cell type) with 40 mM of pH 6.1 PIPES buffer, 25 or 40 mM lactate supplementation, and a combination of both. In all CFS the consumption rate of phosphocreatine remained unaffected by variations in pH or lactate concentration (Supplementary Fig. S3). Table 1 shows the rates of glucose, pyruvate and lactate evolutions in the four conditions for the four tested CFS. The corresponding raw time series are presented in Supplementary Figs. 4–7.

**Table 1:** Rates of glucose (G), pyruvate (P) and lactate (L) evolutions in µM/min for HEK, HeLa, reticulocyte and INS-1E CFS in control conditions (pH 6.9–7.5), acidic conditions (pH 6.5–6.8), and high lactate concentration condition, either at control (pH 6.9–7.6, 40 mM added lactate) or acidic conditions (pH 6.4–6.9, 40 or 25 mM added lactate).

	HEK	HeLa	Reticulocyte	INS-1E
Control pH: 6.9–7.5	G: [− 26.5; − 12.1]	G: [− 25.9; − 18.8]	G: [− 23.2; − 11.7]	G: [NS; 1]
	L: [16.2 27.6]	L: [13.9; 22.1]	L: [1.4; 1.4]	L: [0.5; 1.7]
	P: [NS; 0.4]	P: NS	P: [NS; 0.3]	P: [1.3; 1.7]
	n = 3	n = 4	n = 2	n = 2
Acidic pH: 6.4–6.8	G: [− 4.5; − 2.7]	G: − 6.1	G: − 10.0	G: [0.3; 0.8]
	L: [− 0.2; 1.3]	L: 0.64	L: 0.43	L: [− 0.7; − 0.2]
	P: [− 0.2; NS]	P: ND	P: − 0.79	P: [0.1; 0.4]
	n = 3	n = 1	n = 1	n = 2
Control pH: 6.9–7.6	G: [− 27.8; − 14.6]	G: − 19.1	G: [− 9.0; − 6.2]	G: [NS; 0.4]
Lactate: 40 mM	L: [7.2; 14.7]	L: 24	L: [1.6; 6.4]	L: [− 6.1; − 1.4]
	P: [0.4; 1.4]	P: 1.6	P: NS	P: [0.5; 0.8]
	n = 3	n = 1	n = 2	n = 2
Acidic pH: 6.4—6.9	G: [− 4.9; − 3.3]	G: [− 5.3; − 2.7]	G: − 4.1	G: [0.09; 1.2]
Lactate: 40 mM (HeLa, INS-1E, reticulocyte) or 25 mM (HEK)	L: [NS; 7.2]	L: [− 5.6; NS]	L: NS	L: [− 2.3; − 1.6]
	P: [− 0.09; NS]	P: [NS; 0.5]	P: − 0.35	P: [NS; 0.6]
	n = 3	n = 2	n = 1	n = 2

The number of replicates is indicated in each box. For replicated measures, rates are displayed as [minimal observed value; maximal observed value]. Non-detected metabolites are reported as ND. Non-significant evolutions are reported as NS and are the results of Pearson’s or Spearman’s tests for the correlation between time and the compound concentration. Negative or positive rates respectively indicate that the compounds are consumed or produced.

HEK and HeLa CFS presented equivalent behaviours in all tested conditions. In control conditions they presented similar glucose consumption rates (12.1–26.5 µM/min and 18.8–25.9 µM/min respectively), similar lactate production rates (16.2–27.6 µM/min and 13.9–22.1 µM/min respectively), and pyruvate concentrations that were both constant, but different (0.02 mM and 0.05 mM respectively, Supplementary Figs. S4 and S5). Acidic pH drastically decreased glucose consumption rates down to 2.7 µM/min for HEK CFS and 6.1 µM/min for HeLa CFS, repressed lactate production (lactate evolved at a maximal rate of 1.3 µM/min for HEK, and 0.64 µM/min for HeLa CFS), and could cause a slow pyruvate decrease in HEK CFS, at a maximal rate of 0.2 µM/min, while pyruvate was not detected in HeLa CFS. At a high lactate concentration, neither HEK nor HeLa showed changes in their glucose consumption rates, respectively 14.6–27.8 µM/min and 19.1 µM/min. HEK presented a lower lactate production (7.2–14.7 µM/min) whereas HeLa showed a slight increase in the lactate production rate (24 µM/min), and they both had higher pyruvate production rates, 0.4–1.4 µM/min for HEK and 1.6 µM/min for HeLa CFS. Combined acidic pH and 40 mM lactate concentration affected glucose consumption and lactate production similarly to acidic pH, with glucose decreasing at a maximal rate of 4.9 µM/min for HEK and 5.3 µM/min for HeLa CFS, and lactate evolving at a rate of 0–7.2 µM/min for HEK and (− 5.6)–0 µM/min for HeLa CFS. For HEK CFS, pyruvate was maintained constant, but interestingly at a higher concentration than in control and low pH conditions (0.04 mM versus 0.02 mM, Supplementary Fig. S4), which was not observed in HeLa CFS where pyruvate concentration remained at 0.05 mM as in control and acidic pH conditions (Supplementary Fig. S5).

Reticulocyte CFS presented similar reactions to the tested conditions as HEK and HeLa CFS. In control conditions the glucose consumption rate was similar to the one of HEK and HeLa CFS (11.7–23.2 µM/min), lactate production was slower than for HEK and HeLa CFS (1.4 µM/min), and pyruvate concentration was constant but about 10 times higher than in HEK and HeLa CFS, at 0.3 mM (Supplementary Fig. S6). In acidic pH conditions, glucose consumption slowed down to 10 µM/min, lactate production down to 0.43 µM/min and pyruvate started to be consumed at a rate of 0.79 mM/min. At 40 mM lactate concentration compared to the control, glucose consumption slowed down to 6.2 µM/min, lactate production increased to 6.4 µM/min and pyruvate remained constant. Compared to acidic pH, combined acidic pH and 40 mM lactate concentration slowed glucose consumption down to 4.1 µM/min, made lactate production undetectable, and caused a slower pyruvate consumption of 0.35 µM/min.

INS-1E CFS showed a very different behaviour compared to HEK, HeLa and reticulocyte CFS. In control conditions they did not consume glucose that was either constant or produced at a very slow rate of 1 µM/min, they performed low-rate (0.5–1.7 µM/min) lactate production, showed a 3 (compared to reticulocyte CFS) to 50 (compared to HEK CFS) times higher initial pyruvate concentration of 1 mM, and a 1.3–1.7 µM/min pyruvate accumulation (Supplementary Fig. S7 and Table 1). In all other conditions compared to the control, glucose consumption was unaltered with evolution rates varying insubstantially between 0 and 1.2 µM/min, lactate production was reversed to a lactate consumption, and pyruvate accumulation was decreased similarly within the range of 0–0.8 µM/min. Lactate consumption was the slowest in the acidic pH condition, with a rate of 0.2–0.7 µM/min, and the highest in conditions of 40 mM lactate concentration, with a rate of 1.4–6.1 µM/min in control pH, and of 1.6–2.3 µM/min in acidic pH.

In summary, acidification slowed the glucose consumption rate in various phenotypes of lactic fermentation (HEK, HeLa and reticulocyte CFS); it inhibited lactate production in all tested CFS types and even reversed it to a lactate consumption in INS-1E CFS. High lactate concentration had no significant impact on the lactic fermentation phenotypes apart from triggering pyruvate accumulation in cancer ones (HEK and HeLa CFS). In the INS-1E CFS, contrastingly, high lactate concentration reversed lactate production to a consumption. When combined, low pH and high lactate concentration intensified lactate consumption in the INS-1E CFS. In the CFS showing lactic fermentation they affected glucose consumption and lactate production in a similar fashion as low pH conditions, and they elevated pyruvate concentration in HEK CFS in which it was the lowest of all CFS in control conditions.

## Discussion

In this study, our aim was to characterise the glucose catabolism properties of cytosols originating from cells with different phenotypes of cancer and lactic fermentation, in response to direct acidification and lactate accumulation. As an example, in HEK CFS, glucose was consumed over time while lactate was produced, which evidenced and characterised an active glycolytic pathway. Those CFS presented the enzymatic content required to address the impact of acidification and high lactate concentration on glucose catabolism and lactic fermentation. RT-NMR was a method of choice that enabled the monitoring of several glucose-derived metabolites in controlled conditions of ATP, glucose, pH and lactate. Our approach, consisting of examining enzymatic reactions within reactivated CFS using RT-NMR, represents a solid experimental strategy for investigating metabolic events and their regulation. Its simplified and versatile setup is easily customisable, and thus well-suited to screening experiments. Moreover, by using widely accessible 1H-NMR spectrometers, the acquisition time of a dataset was approximately 90 min. This makes our approach amenable to medium-throughput applications in metabolomics.

The four CFS assessed in this study originated from a diversity of cancer and lactic fermentation phenotypes. HEK and HeLa CFS displayed the characteristics of a cancer Warburg-type metabolism: we showed that they harboured HK-1 and HK-2 isoforms, which are upregulated in cancer^17^, high amounts of LDH-A and -B subunits^16^, and a high rate of protein translation, glucose consumption and lactate production. Interestingly, although HK-1 and HK-2 are known to be bound to mitochondria in cells^17^, the CFS preparation protocol allowed to recover them in the cytosolic fractions, be they homemade (HEK CFS) or commercial (HeLa CFS). Reticulocyte CFS, in line with previous reports^26–28^, performed lactic fermentation at a much slower rate than HEK and HeLa cells. They presented abundant LDH-B and no detectable LDH-A, consistently with a non-cancer phenotype and a low-rate lactic fermentation. INS-1E cells are an exception in the metabolic phenotypes of cancer as they do not perform lactic fermentation^24,34^. This characteristic was confirmed in the INS-1E CFS that contained the HK-4 isoform of HK with the lowest affinity for glucose (Km = 8 mM^35^), and consistently did not show a detectable glucose consumption in our conditions (1 mM glucose).

We evidence that acidification has a major impact on glucose catabolism in all tested CFS except INS-1E, where no impact on glucose evolution was detected compared to control. In HEK, HeLa and reticulocyte CFS, lowering pH to 7.0 instantly slowed glucose consumption down to 25% of its control rate, which makes our results in CFS consistent with observations in differently prepared cell lysates and in whole cells over similar short timescales^13,36^. These observations also confirm the well-reported general inhibition of glycolysis enzymes activities by low pH^13,37^. Similar results observed over the HEK, HeLa and reticulocyte CFS suggest that the inhibitory effect of acidification is widespread across lactic fermentation phenotypes. Moreover, the undetectable lactate production suggests that glucose carbons are redirected to other pathways than lactic fermentation.

We also show that cytosolic acidification instantly inhibits lactate production, in accordance with observations in cell lysates and in whole cells^13,38^. Acidification has been shown to regulate LDH activity by altering its spatial conformation^38–40^. We observed acidification-induced inhibition of lactate synthesis in cytosols containing both LDH-A and -B subunits or only LDH-B. This tends to indicate that the inhibition of lactate production is not LDH-isoform-specific. Reversely, we observed that despite a similar LDH-B isoform content, INS-1E CFS consumed lactate at low pH, unlike reticulocyte cytosols. The way INS-1E and reticulocyte CFS processed lactate was thus not correlated to the LDH isoforms they contained, but rather to their ability to consume glucose. It is important to note that INS-1E cells harbour the low glucose-affinity isoform HK4, also known as glucokinase (GCK). Consequently, we can not draw definitive conclusions about the impact of pH and lactate concentration on GCK-mediated glycolysis in INS-1E cells. Activating glycolysis in the context of our study would have required higher glucose levels than those employed for other CFS. Such high glucose concentrations would have placed us under entirely different conditions compared to the other CFS setups we utilised, and we chose to maintain consistency for meaningful comparisons.

Moreover, we found that in CFS performing lactic fermentation, the presence of a high lactate concentration did not alter its production, contrary to INS-1E CFS for which lactate supplementation induced its consumption, in line with previous reports and the physiological function of pancreatic β-cells^41^. The absence of effects due to lactate supplementation was thus not related to the specific expression of LDH-A, although known to have a lower affinity for lactate than LDH-B^42–44^, but more generally to the lactic fermentation phenotype. It shows that the single LDH expression pattern does not sufficiently explain cytosol’s ability to consume lactate, as an exclusive presence of LDHB is necessary but not sufficient to observe a lactate-to-pyruvate conversion (in INS-1E vs. reticulocyte CFS). Together with Xie et al., our results confirm a differential processing of lactate between whole cells and isolated cytosols. Whole cells under lactic acidosis perform lactate consumption^13,45^, which is not observed in isolated cytosolic fractions, suggesting that lactate would be routed to other organelles such as mitochondria^46^.

Interestingly, we show that increasing lactate concentration raised the pyruvate concentration only in cancer Warburg-type CFS (HEK and HeLa). In such cytosols submitted to combined low pH and high lactate concentration, pyruvate concentration was higher than in control conditions. The underlying mechanisms are not clear. Acidity and high lactate concentration could both alter pyruvate homeostasis, by modulating the LDH equilibrium constant for pH, and the LDH reaction quotient for lactate concentration. Notwithstanding, the resulting higher pyruvate concentration in the cytosol may increase its availability for other organelles. It has notably been shown that lactate and lactic acidosis increase the importance of mitochondria in the process of glucose^7^.

Apart from its relatively minor impact on pyruvate homeostasis, lactate alone had no clear direct impact on lactic fermentation in Warburg-type CFS even in supraphysiological concentration. However, lactate has been shown to deeply impact cancer cells' physiology, notably by promoting and sustaining an oxidative phenotype in cancer^47–49^. Our results suggest that the impact of lactate on the cytosolic catabolism of glucose in cancer cells relies on alternative regulation processes such as the alteration of proton exchanges at the plasma membrane^7^, signalling^50^ or epigenetic modulations^51^, that are not exclusive to the cytosol and occurs on longer timescales. Contrastingly, our findings suggest that the metabolic alteration induced by acidosis in cancer^52–56^ involves a direct interaction of protons with the cytosolic machinery of glucose catabolism, and can be observed almost instantly in isolated cytoplasmic fractions.

In summary, our results along with the cited works support the view that in cancer lactic acidosis, acidification acts as a strong regulator on cell metabolism, by directly downregulating the Warburg effect^12,18^, while lactate acts as a later regulator to reactivate alternative catabolic pathways, such as the oxidative metabolism of mitochondria^7^. This view has successfully yielded recent therapy strategies targeting oxidative phosphorylation, which is necessary to cancer cells in acidic contexts^57,58^. Our approach, which relies on the examination of enzymatic reactions within a reactivated CFS using RT-NMR, represents a solid experimental strategy for investigating metabolic events and their regulation within a controlled sample. This versatile setup can be easily customised for conditions and drug testing, and amenable to medium-throughput applications in drug screening, toxicity assessment and pharmaceutical projects. Our study supports this view with a novel method that is complementary to the ones commonly used in cancer research.

In summary, our results along with the cited works support the view that in cancer lactic acidosis, acidification acts as a strong regulator of cell metabolism, by directly downregulating the Warburg effect^12,18^, while lactate acts as a later regulator to reactivate alternative catabolic pathways, such as the oxidative metabolism of mitochondria^7^. This perspective has recently led to the development of therapeutic strategies targeting oxidative phosphorylation, a critical process for cancer cells in acidic environments^57,58^. Our study contributes to this understanding with a novel approach, centred on the analysis of enzyme reactions within a reactivated cell-free system using real-time nuclear magnetic resonance (RT-NMR), which offers a robust experimental method for investigating metabolic processes and their control in a controlled setting.

### Supplementary Information

Supplementary Information.

## Data Availability

The datasets acquired and analysed during the current study are available from the corresponding author on reasonable request.
