# Supplementary material for: Warburg-associated acidification represses lactic fermentation independently of lactate, contribution from real-time NMR on cell-free systems
Source: Sci Rep. 2023 Oct 18;13:17733. doi: 10.1038/s41598-023-44783-3 (PMC10584866; doi:10.1038/s41598-023-44783-3)
Supplement: Supplementary file 1 — Supplementary Information. [file 41598_2023_44783_MOESM1_ESM.docx]

**SUPPLEMENTARY FILE**

**Title :** Warburg-associated acidification represses lactic fermentation independently of lactate, contribution from Real-Time NMR on cell-free systems

**Authors & affiliations:**

Zoé Daverio^1,2,&^ , Maxime Kolkman^1,3,&^, Johan Perrier^1,&^ , Lexane Brunet^1^, Nadia Bendridi^1^, Corinne Sanglar^4^, Marie-Agnès Berger^1^, Baptiste Panthu^1,†,*^ and Gilles JP Rautureau^3,†,*^

^1^ Laboratoire CarMeN, UMR INSERM U1060/INRAE U1397, University of Lyon, Université Claude Bernard Lyon 1, F-69310 Pierre-Bénite, France

^2^ Master de Biologie, École Normale Supérieure de Lyon, University of Lyon, Université Claude Bernard Lyon 1, 69342 Lyon Cedex 07, France

^3^ Institut de Chimie et Biochimie Moléculaires et Supramoléculaires, ICBMS UMR 5246, University of Lyon, Université Claude Bernard Lyon 1, 69622 Lyon, France;

^4^ Institut des Sciences Analytiques, UMR5280 CNRS, University of Lyon, Université Claude Bernard Lyon 1, 5 rue de la Doua, Villeurbanne 69100, France

^&^ First co-authors

^†^ Last co-authors

^*^ Correspondence: [gilles.rautureau@univ-lyon1.fr](mailto:gilles.rautureau@univ-lyon1.fr); [baptiste.panthu@univ-lyon1.fr](mailto:baptiste.panthu@univ-lyon1.fr)

[zoe.daverio-feige@ens-lyon.fr](mailto:zoe.daverio-feige@ens-lyon.fr)

[maxime.kolkman@student.uliege.be](mailto:Maxime.Kolkman@student.uliege.be)

[johan.perrier.mailpro@gmail.com](mailto:johan.perrier.mailpro@gmail.com)

[lexane.brunet@etu.univ-lyon1.fr](mailto:lexane.brunet@etu.univ-lyon1.fr)

[corinne.sanglar@isa-lyon.fr](mailto:corinne.sanglar@isa-lyon.fr)

[nadia.bendridi@inserm.fr](mailto:nadia.bendridi@inserm.fr)

[marie-agnes.berger@inserm.fr](mailto:marie-agnes.berger@inserm.fr)

[baptiste.panthu@univ-lyon1.fr](mailto:baptiste.panthu@univ-lyon1.fr)

[gilles.rautureau@univ-lyon1.f](mailto:gilles.rautureau@univ-lyon1.fr)r

ORCID iD:

Rautureau GJP: 0000-0002-1064-0293

Panthu B: 0000-0001-9430-4061

**SUPPLEMENTARY FIGURES**


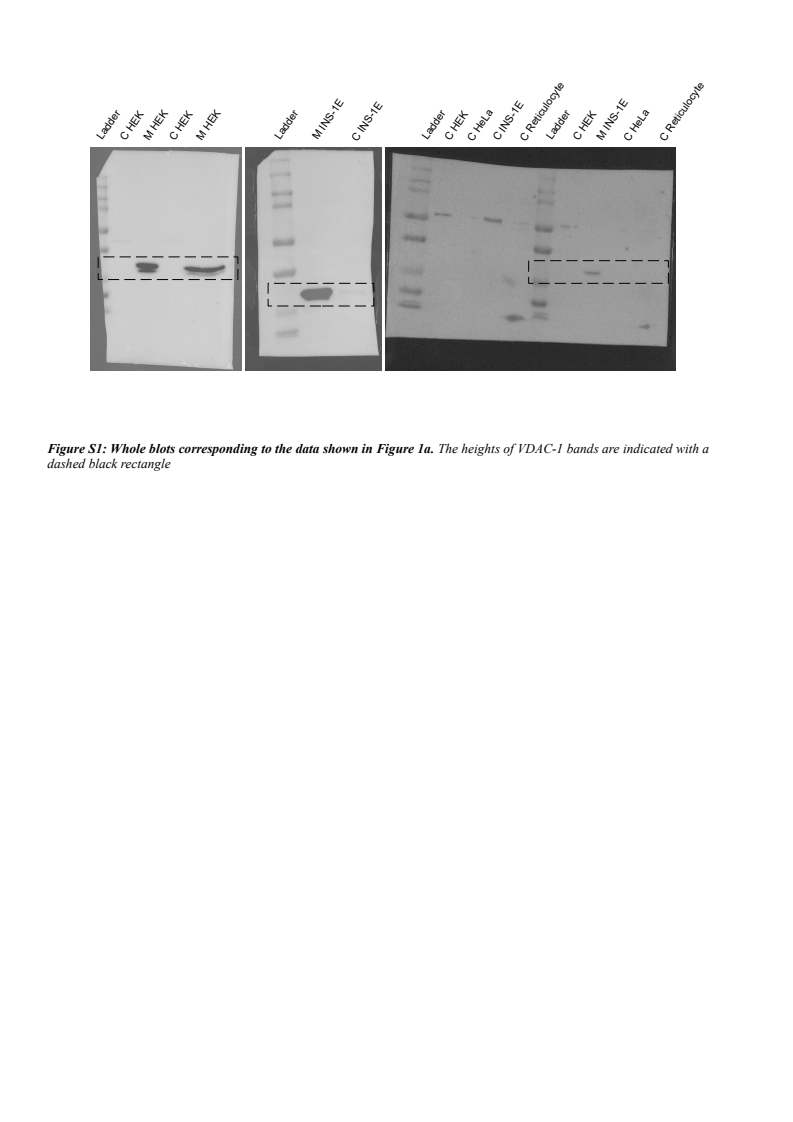
***Figure S1: Whole blots corresponding to the data shown in Figure 1a.*** *The heights of VDAC-1 bands are indicated with a dashed black rectangle*


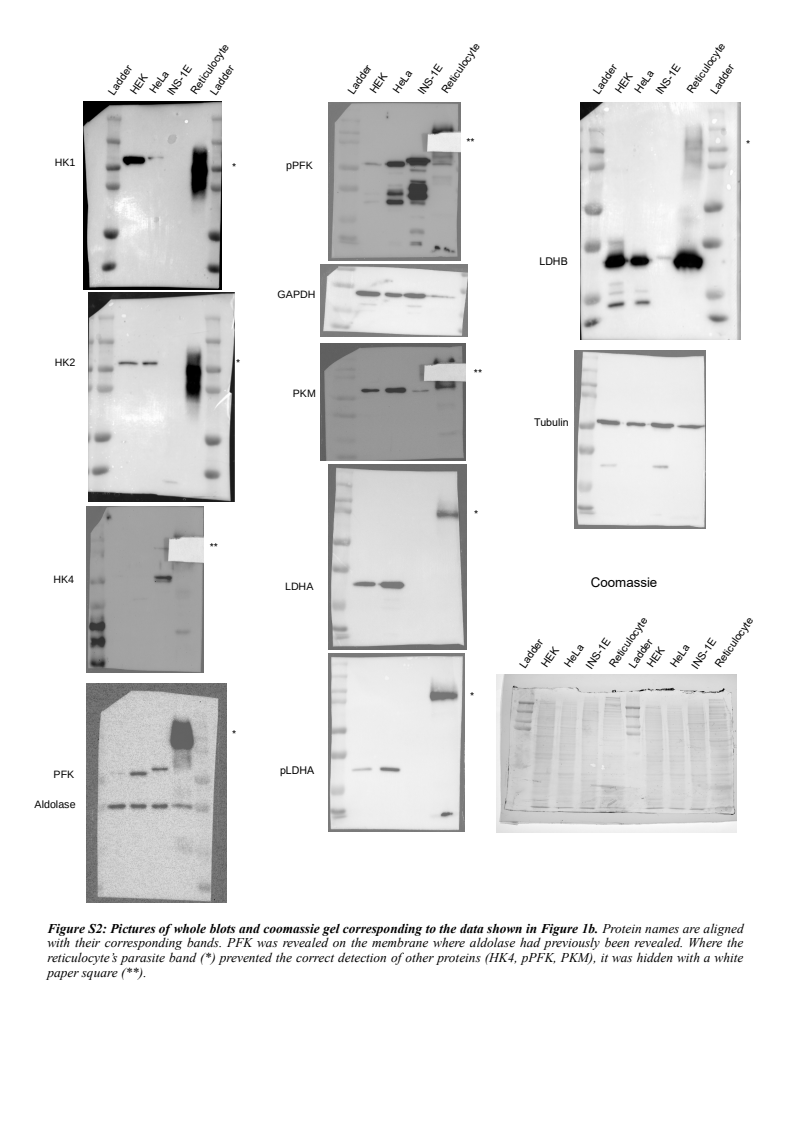
***Figure S2: Pictures of whole blots and coomassie gel corresponding to the data shown in Figure 1b.*** *Protein names are aligned with their corresponding bands. PFK was revealed on the membrane where aldolase had previously been revealed. Where the reticulocyte’s parasite band (*) prevented the correct detection of other proteins (HK4, pPFK, PKM), it was hidden with a white paper square (**).*


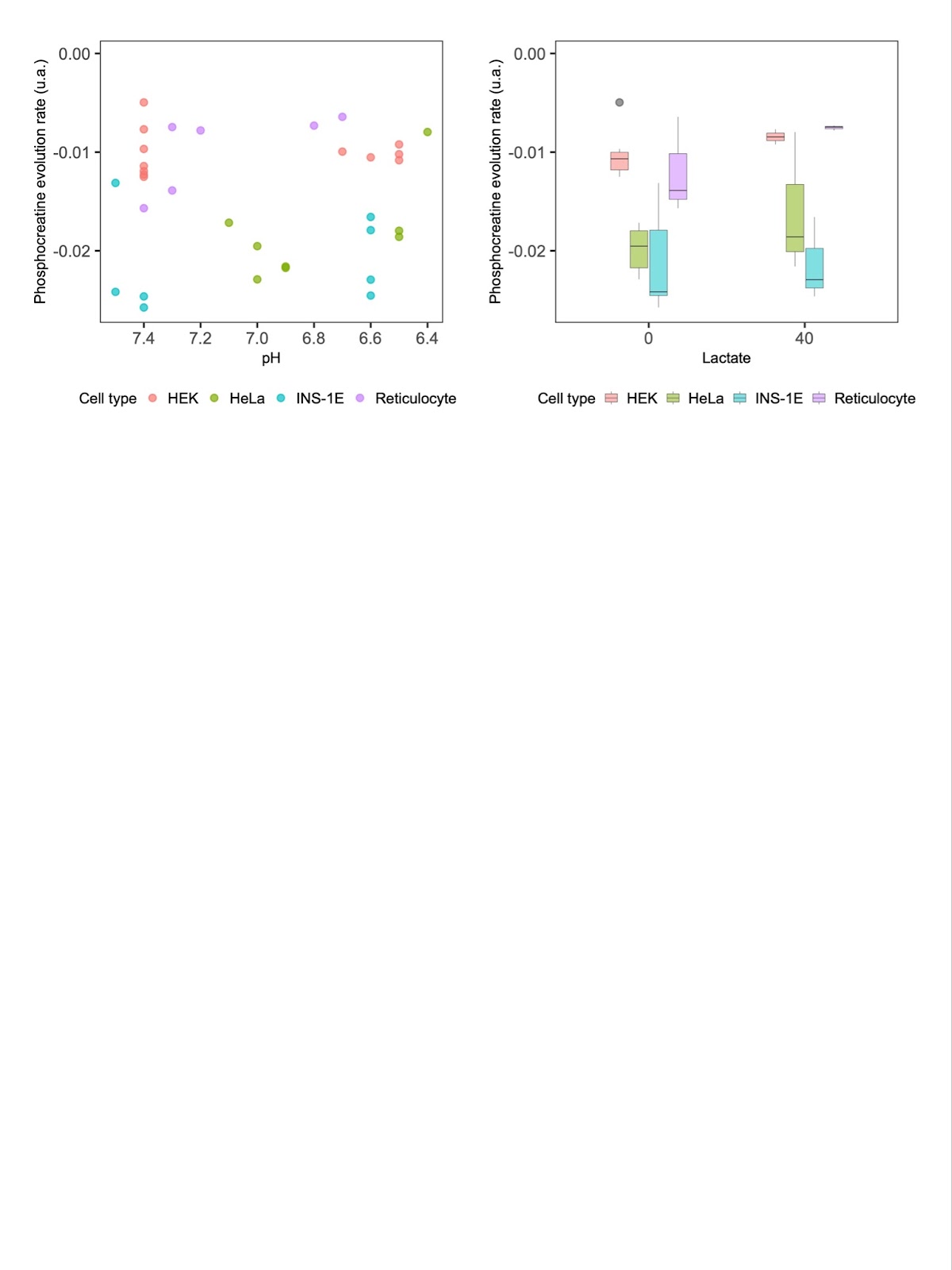


***Fig. S3 : Phosphocreatine evolution rate is not affected by pH or lactate concentration in HEK, HeLa, reticulocyte and INS-1E CFS.*** *Phosphocreatine evolution rate in arbitrary units in HEK, HeLa, reticulocyte and INS-1E CFS at pH 6.4-7.6 (left panel) and at 0 or 40 mM added lactate (right panel).*


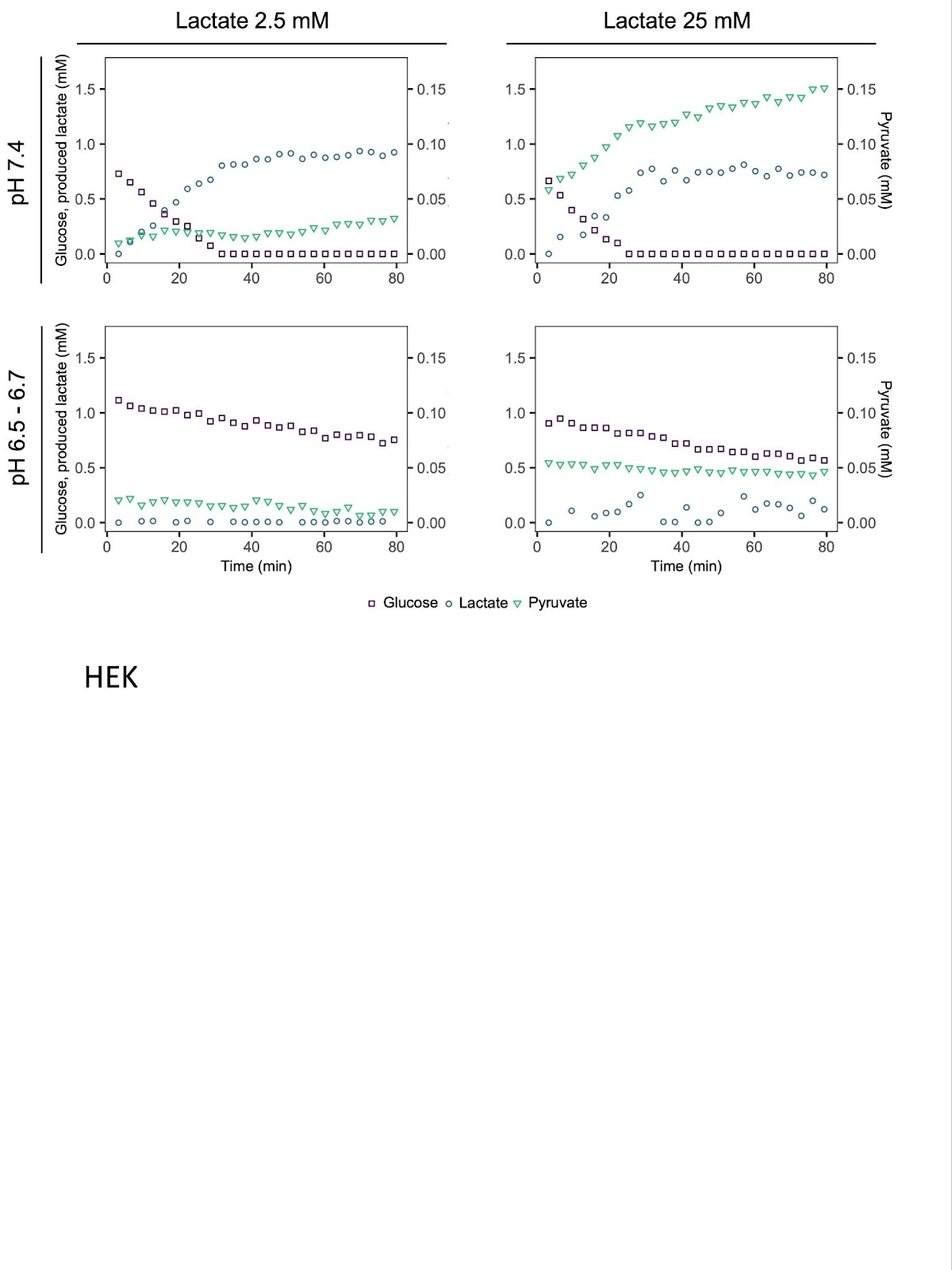
***Figure S4: Combined effects of low pH and lactate supplementation on the glucose catabolism of HEK CFS.*** *Simultaneous* *evolutions of glucose (*
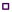
*), lactate newly produced in the time course of the experiment (*
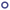
*) and pyruvate (*
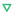
*) in HEK CFS in the control condition (pH 7.4, 2.5 mM lactate intrinsic to the CFS), low pH conditions (pH 6.5-6.7), 25 mM lactate supplementation at a control pH of 7.4, and a condition of low pH (pH 6.5-6.7) and 25 mM lactate supplementation for two replicate CFS. For each graph the left axis indicates the concentrations of glucose and newly produced lactate, and the right axis indicates the pyruvate concentration.*


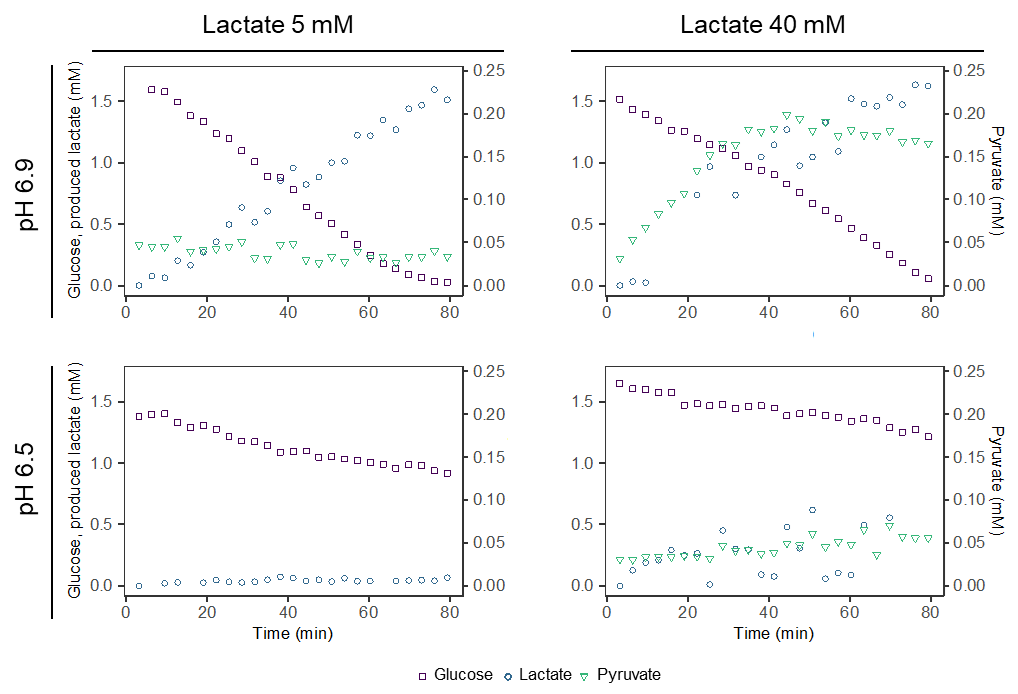
***Figure S5: Combined effects of low pH and lactate supplementation on the glucose catabolism of HeLa CFS.*** *Simultaneous* *evolutions of glucose (*
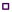
*), lactate newly produced in the time course of the experiment (*
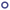
*) and pyruvate (*
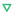
*) in HeLa CFS in the control condition (pH 6.9, 5 mM lactate intrinsic to the CFS), low pH conditions (pH 6.5) in which pyruvate was not detectable, 40 mM lactate supplementation at a control pH of 6.9, and a condition of low pH (pH 6.5) and 40 mM lactate supplementation. For each graph the left axis indicates the concentrations of glucose and newly produced lactate, and the right axis indicates the pyruvate concentration.*


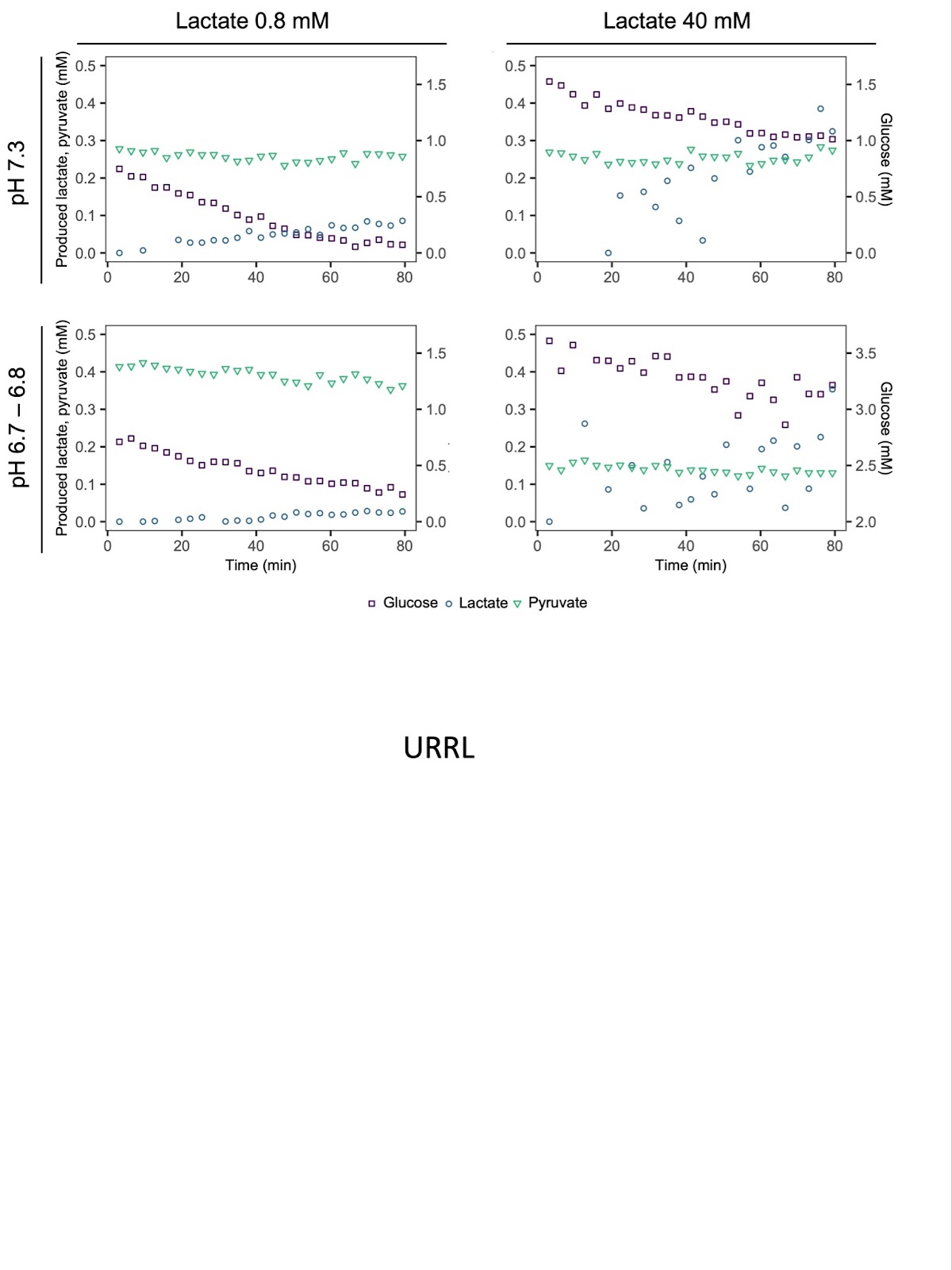
***Figure S6: Combined effects of low pH and lactate supplementation on the glucose catabolism of reticulocyte CFS.*** *Simultaneous* *evolutions of glucose (*
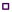
*), lactate newly produced in the time course of the experiment (*
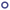
*) and pyruvate (*
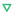
*) in reticulocyte CFS in the control condition (pH 7.3, 0.8 mM lactate intrinsic to the CFS), low pH conditions (pH 6.7-6.8), 40 mM lactate supplementation at a control pH of 7.3, and a condition of low pH (pH 6.7-6.8) and 40 mM lactate supplementation. For each graph the left axis indicates the concentrations of pyruvate and newly produced lactate, and the right axis indicates the glucose concentration.*


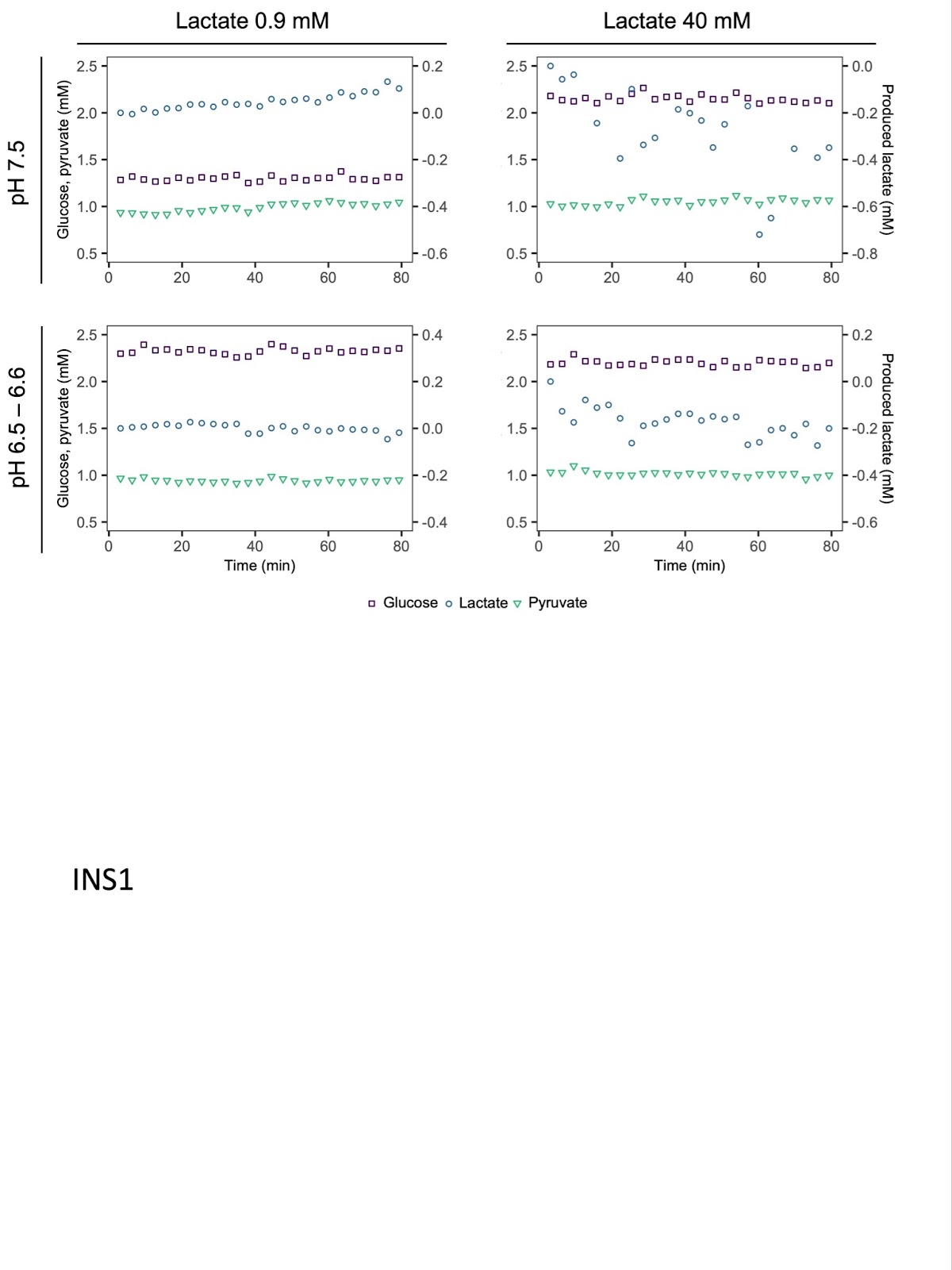
***Figure S7: Combined effects of low pH and lactate supplementation on the glucose catabolism of INS-1E CFS.*** *Simultaneous* *evolutions of glucose (*
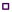
*), lactate newly produced in the time course of the experiment (*
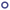
*) and pyruvate (*
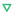
*) in INS-1E CFS in the control condition (pH 7.5, 0.9 mM lactate intrinsic to the CFS), low pH conditions (pH 6.5-6.6), 40 mM lactate supplementation at a control pH of 7.5, and a condition of low pH (pH 6.5-6.6) and 40 mM lactate supplementation. For each graph the left axis indicates the concentrations of glucose and pyruvate, and the right axis indicates the newly produced lactate concentration.*

**SUPPLEMENTARY TABLES**

**Table S1:** Characteristics of HEK, HeLa, INS-1E and reticulocyte CFS.

| CFS | Commercial/ homemade | Cancer cell | Lactic fermentation | Initial pH in control conditions | Initial pH in acidic conditions |
| --- | --- | --- | --- | --- | --- |
| HEK | Homemade | Yes | Yes | 7.4 – 7.5 | 6.5 |
| HeLa | Commercial | Yes | Yes | 6.9 | 6.5 |
| Reticulocyte | Commercial | No | Yes | 7.3 | 6.7-6.8 |
| INS-1E | Homemade | Yes | No | 7.5 | 6.5-6.6 |
